# Nanoliter Centrifugal Liquid Dispenser Coupled with Superhydrophobic Microwell Array Chips for High-Throughput Cell Assays

**DOI:** 10.3390/mi9060286

**Published:** 2018-06-06

**Authors:** Yuyi Wang, Yushuai Wu, Yue Chen, Jianxiong Zhang, Xiaofang Chen, Peng Liu

**Affiliations:** 1 Department of Biomedical Engineering, School of Medicine, Collaborative Innovation Center for Diagnosis and Treatment of Infectious Diseases, Tsinghua University, Beijing 100084, China; yy-wang15@mails.tsinghua.edu.cn (Y.W.); wu-ys17@mails.tsinghua.edu.cn (Y.W.); yue-c14@mails.tsinghua.edu.cn (Y.C.); zhangjx13@mails.tsinghua.edu.cn (J.Z.); 2 School of Biological Science and Medical Engineering, Beihang University, Beijing 100191, China; xfchen@buaa.edu.cn

**Keywords:** nanoliter liquid dispensing, superhydrophobicity, microwell array, high-throughput screening, drug screening, microfluidics

## Abstract

Microfluidic systems have been regarded as a potential platform for high-throughput screening technology in drug discovery due to their low sample consumption, high integration, and easy operation. The handling of small-volume liquid is an essential operation in microfluidic systems, especially in investigating large-scale combination conditions. Here, we develop a nanoliter centrifugal liquid dispenser (NanoCLD) coupled with superhydrophobic microwell array chips for high-throughput cell-based assays in the nanoliter scale. The NanoCLD consists of a plastic stock block with an array of drilled through holes, a reagent microwell array chip (reagent chip), and an alignment bottom assembled together in a fixture. A simple centrifugation at 800 rpm can dispense ~160 nL reagents into microwells in 5 min. The dispensed reagents are then delivered to cells by sandwiching the reagent chip upside down with another microwell array chip (cell chip) on which cells are cultured. A gradient of doxorubicin is then dispensed to the cell chip using the NanoCLD for validating the feasibility of performing drug tests on our microchip platform. This novel nanoliter-volume liquid dispensing method is simple, easy to operate, and especially suitable for repeatedly dispensing many different reagents simultaneously to microwells.

## 1. Introduction

High-throughput cell-based assays have become an indispensable workhorse for discovering new drug candidates [1,2], deciphering complicated cellular processes [3,4], annotating gene functions [5], etc. Nowadays, these large-scale experiments highly depend on various robotic workstations to realize automated and high-throughput operations [1]. Although an unprecedented throughput has been achieved based on conventional 384- or even 1536-well microtiter plates coupled with robotic liquid handling, this well-established working scheme is approaching its physical limitation in terms of the throughput [6,7]. In addition, the expensive robotic equipment, together with the consumptions of large amounts of consumables and valuable reagents, make the assays unaffordable for common laboratories with limited budgets, therefore restricting the broad application of the high-throughput systems. It would seem the pursuit of higher throughputs and lower costs for cell-based assays is inevitably leading to the miniaturization of reaction volumes by which more assays can be arranged and fewer reagents are needed.

Microfabrication technology has great potential to shrink the reaction volume down to the nanoliter scale owing to its capability of fabricating high-density microstructures [7,8]. According to the way they manipulate fluids, microfabricated platforms for high-throughput cell-based analyses can be categorized into three working modes: perfusion flow mode, droplet mode, and microarray mode [7]. Perfusion flow microsystems usually utilize microchannels to manipulate cells and deliver reagents [9,10,11,12]. Although the operation is convenient once the required pumping and tubing system is set up, the complicated microchannel networks limit its throughput to a few hundred assays in parallel. Droplet mode systems employ water-in-oil emulsion droplets for high-throughput cell analyses [13,14,15]. The major advantage of the droplet system is the incomparable throughput, with millions of droplets able to be easily generated and analyzed. However, the flowing droplets are hard to track for long-term analyses and are not suitable for multi-step assays. In contrast to the perfusion flow and the droplet modes, microarray-based systems usually contain thousands of microwells or microislands fabricated by photolithography on a 2D planar substrate [16,17,18,19]. Cells cultured within these stationary structures can be tracked for a long period of time and examined with a variety of cell analytical methods [7]. While these microarray devices can substitute the conventional microtiter plates, the handling of small-volume liquid in these devices still relies on expensive and bulky robotic equipment. For example, microarray spotters with either contact printing needles or non-contact piezoelectric nozzles are often employed to dispense nanoliter reagents to microwells [20,21,22]. Unfortunately, as the types of reagents that require the pipetting increase, this liquid dispensing process becomes extremely long. This is because the reusable dispensers need to be washed thoroughly to prevent cross-contamination each time the reagent is changed. It only takes a few minutes to deliver one reagent to 100 wells, but it may take hours to deliver 100 reagents to 100 microwells with a single dispenser, with washing taking up a large portion of the time. Several new tools have been developed for dispensing nanoliter-volume reagents in parallel [23]. For example, Koltay et al. demonstrated a novel dispensing well plate that consists of up to 1536 dispensing units for high-throughput reagent delivery [24]. Focused acoustics was successfully employed to inject droplets from a reagent source plate to an assay plate in a non-contact way, eliminating the time-consuming wash step [25,26]. Similarly, Zhou et al. developed a polydimethylsiloxane (PDMS) microfluidic pipette chip to deliver reagents to openly accessible microwells [27]. While these methods are extraordinary in throughput, they still need the assistance of precise translational platforms, and the costs of these dispensing systems may remain high.

Previously, our group had successfully developed a novel superhydrophobic microwell array chip (SMARchip) for high-throughput cell-based analysis [22]. By grafting a thick layer of superhydrophobic polymers onto the top surface of a PDMS microwell array, the conditions in individual microwells were completely isolated due to the repelling effect of the polymers to the aqueous solution. The imaging-based investigation of stem cell niches as well as the high-throughput *in situ* cell electroporation demonstrated by our group proved this platform has great potential to significantly update high-throughput cell screening studies [22,28]. However, like most microarray systems, the use of a microarray spotter for liquid dispensing is a bottleneck in the operations of the SMARchip. Therefore, we present here a novel nanoliter centrifugal liquid dispenser (NanoCLD) that utilizes centrifugal force to deliver reagents from a stock block to a superhydrophobic microwell reagent chip in parallel. By aligning this reagent chip upside down to another SMARchip for cell culture, these reagents can be introduced to cells in a high-throughput manner. As an ordinary centrifuge is the only equipment needed for liquid dispensing, this method is inexpensive, easy to operate, and suitable for common laboratories that do not have access to high-end robotic workstations to perform high-throughput cell assays.

## 2. Materials and Methods

### 2.1. Superhydrophobic Microwell Array Chip

**Figure 1:**
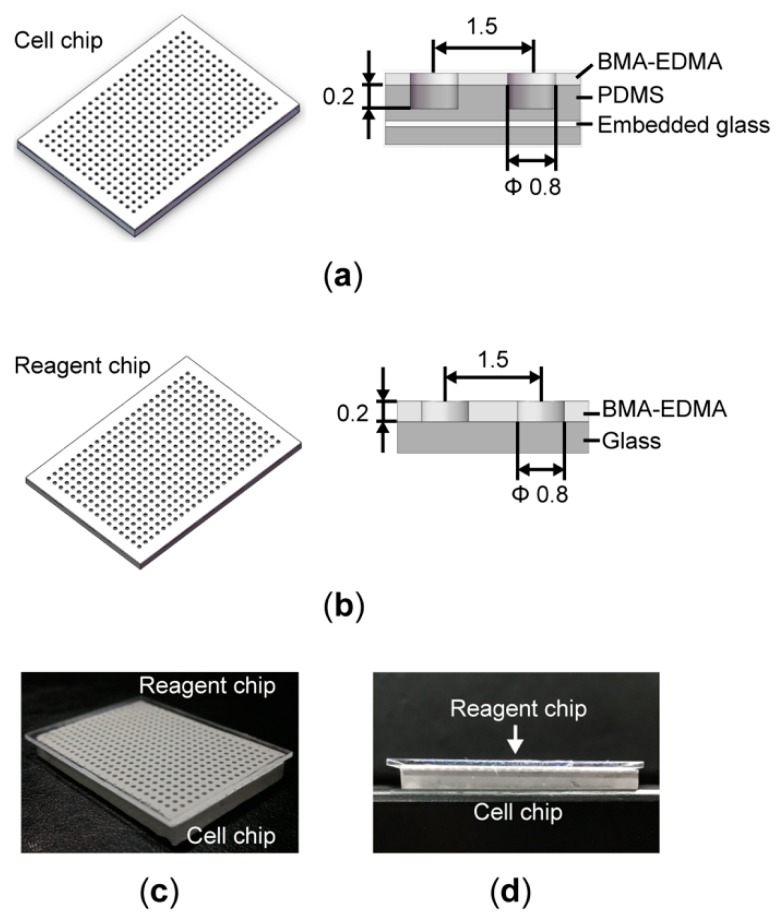
High-throughput superhydrophobic cell culture system. (**a**) Schematic of the cell chip with a 384-microwell array. The cross-sectional view of the chip shows the polydimethylsiloxane (PDMS) microwell and has a diameter of 0.8 mm, a depth of 0.2 mm, and a spacing of 1.5 mm. A thick layer of superhydrophobic polymers (BMA-EDMA) is attached to the top surface of the microwell array. A piece of glass coverslip is embedded into the PDMS as a support frame to prevent shrinkage of the microwell array. (**b**) Schematic of the reagent chip. The cross-sectional view of the chip shows a 0.2 mm-thick layer of BMA-EDMA polymers attached to a piece of glass. (**c**) Photo of a reagent chip aligned with a cell chip. (**d**) Photo of a reagent chip sandwiched with a cell chip.

The high-throughput superhydrophobic cell culture system consists of a pair of microwell array chips—a cell chip and a reagent chip. As shown in Figure 1a, the cell chip is made of a microfabricated polydimethylsiloxane (PDMS) microwell array containing 384 wells (800 μm diameter, 200 μm deep, and 700 μm pitch). A ~100-μm-thick superhydrophobic polymer layer (poly(butyl methacrylate-co-ethylene dimethacrylate); BMA-EDMA), which serves as a physical barrier to the culture medium, is synthesized and attached to the top surface of the PDMS microwell array by following the fabrication protocol published previously [22,29]. The corresponding reagent chip shown in Figure 1b is fabricated by attaching a 200-μm-thick layer of BMA-EDMA polymers with 800-μm-diameter holes on a piece of glass. The detailed fabrication protocol can be found in our previous study [28]. Prior to use, both the cell and the reagent chips were soaked in 75% ethanol for 15 min, followed by exposure to Ultraviolet (UV) light for 2 h. As demonstrated in Figure 1c, when these two chips are aligned and pressed together face-to-face, each reagent microwell contacts with the corresponding cell microwell to enable the reagent exchange by diffusion.

### 2.2. Nanoliter Centrifugal Liquid Dispenser

**Figure 2:**
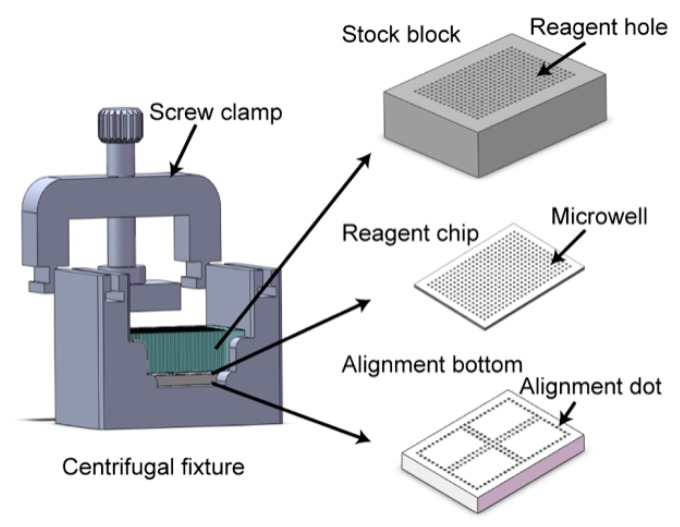
Schematic of the nanoliter centrifugal liquid dispenser (NanoCLD). Assembly of the NanoCLD consisting of four parts: a stock block, a reagent chip, an alignment bottom, and a centrifugal fixture. A screw clamp was employed to hold these parts together in the fixture during centrifugation.

As illustrated in Figure 2, the nanoliter centrifugal liquid dispenser (NanoCLD) comprises of a stock block, a reagent chip, an alignment bottom, and a fixture with a screw clamp. The stock block is a piece of poly(methyl methacrylate) (PMMA) with dimensions of 46 mm × 34 mm × 12 mm, on which 384 holes were drilled through with a pitch of 700 μm. Each 800-μm-diameter reagent hole can hold up to a 5-μL reagent. The upper and the lower surfaces of the stock block were coated with Ultra-Ever Dry^®^ (UltraTech, Jacksonville, FL, USA). Due to the capillary action, the reagents loaded into these reagent holes can stay inside without any leakage. The alignment bottom is also a piece of PMMA with dimensions of 40 mm × 28.5 mm × 6 mm. On the top surface of this, alignment dots were nicked to mark the positions of the reagent holes when the stock block and the alignment bottom aligned together in the fixture.

### 2.3. Procedure of Liquid Dispensing

**Figure 3:**
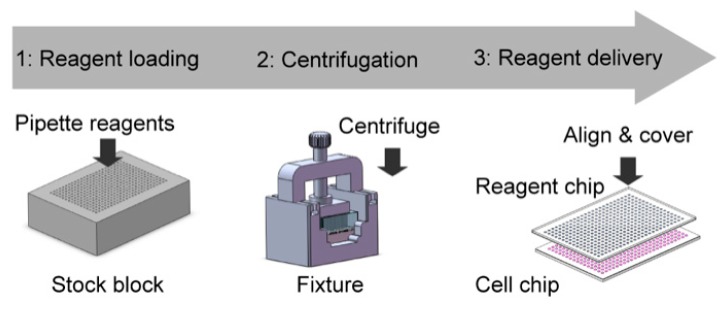
Operating procedure of the NanoCLD. First, reagents were pipetted from 96-plates to the stock block using an adjustable tip spacing pipette. Second, the reagents were dispensed into the reagent chip in the fixture under the centrifugal force. Third, the dispensed reagents were further delivered to cells by aligning and covering the reagent chip upside down onto a cell chip.

The operation procedure of the nanoliter centrifugal liquid dispenser is illustrated in Figure 3 and Supplementary Video S1. Five microliter reagents stored in 96-plates were pipetted into the stock block using an 8-channel electronic pipette with the function of the adjustable tip spacing (VOYAGER II, Integra Biosciences, Hudson, NH, USA). This pipette can adjust the tip spacing from 9 mm to 4.5 mm, which corresponds to the well spacing from a 96- to a 384-plate. As the spacing of the reagent holes in the stock block is 1.5 mm and each row has 24 reagent holes, 24 reagents taken from three rows of a 96-plate can be loaded into one row of the stock block interlaced by three times of pipetting. To assemble the NanoCLD, a reagent chip was first attached to the alignment bottom with a piece of double-sided adhesive tape by aligning the microwells to the corresponding alignment dots. After that, the reagent chip with the alignment bottom and the reagent stock block were put into the aluminum fixture and pressed tightly by turning down the screw clamp. The assembled fixture was then put into a tabletop centrifuge (5810R, Eppendorf AG, Hamburg, Germany) and centrifuged at a speed of 800 rpm for 5 min. As both surfaces between the reagent chip and the stock block were superhydrophobic, no leakage was found during the centrifugation process. After that, the reagent chip with the dispensed reagents in microwells was turned upside down and aligned to the cell chip for the delivery of reagents to cells using both hands with naked eyes or a custom-made microaligner with a stereoscope. This alignment procedure was done in a custom-built glovebox with a controlled humidity to prevent the evaporation of droplets. The alignment and sandwiching operation can be finished within 2 min, which is much less than the time of the reagent evaporation. The stock block with the remaining reagents can be wrapped with Parafilm M (Bemis, Neenah, WI, USA) completely and stored at 4 °C for up to several months without evaporation (shown in Figure S1). When the reagents were used up, the stock block was cleaned with detergents for 15 min in an ultrasonic cleaner, followed by soaking in 75% ethanol for 10 min. After that, the stock block was dried with nitrogen thoroughly for reuse.

Fluorescent dyes, calcein (Coolaber Inc., Beijing, China) and Rhodamine B (Aladdin Bio-Chem, Shanghai, China), were diluted with distilled water (DI water) and delivered into the microwells of the cell chip using the protocol described above to test the performance of the NanoCLD. An inverted microscope (IX83, Olympus, Tokyo, Japan) equipped with a CCD camera (iXon3, Andor, Belfast, Northern Ireland, UK) was used to take images of the microwells. The relative fluorescent units of the images of the microwells with loaded dyes were calculated using the ImageJ software.

### 2.4. On-Chip Cell Culture

MCF-7 cells were cultured in Dulbecco’s modified Eagle’s medium (DMEM) supplemented with 10% fetal bovine serum (FBS), 100 U/mL penicillin, and 100 μg/mL streptomycin (all from Gibco, Grand Island, NY, USA) in a 37 °C incubator with a saturated humidity and 5% CO_2_. Cells were passaged every 2 or 3 days for fewer than 10 passages before abandonment. The detailed operation protocols for on-chip cell culture can be found in our previous study [22,28]. Briefly, prior to cell seeding, the cell chip was coated with Matrigel (BD Biosciences, San Jose, CA, USA) at 37 °C for 2 h. Then, cells were loaded by submerging the entire chip into 1 mL of the cell suspension solution for 15 min to allow the cells to settle, followed by the aspiration of the excess medium. After that, the cell chip was placed in covered double Petri dishes with excess sterile water for cell culture in the incubator. Culture medium was changed every 12 h to provide fresh medium and oxygen to cells. To further verify the on-chip culture, 50 ± 10 MCF-7 cells were loaded into each microwell and cultured for 3 days. Cell viability was assessed at the end of the culture by calcein AM (Invitrogen, Carlsbad, CA, USA) and propidium iodide (PI, Sigma-Aldrich, St Louis, MO, USA) double staining according to the manufacturers’ instructions. As a comparison, cells were also seeded in 24-well plates at a density of 2.5 × 10^4^ cells/cm^2^ as a macroscale control. Cell numbers in 20 randomly picked microwells were counted manually at different time points, whereas the cells in 24-well plates were counted using a hemocytometer.

### 2.5. Drug-Induced Cell Apoptosis

First, 100 ± 10 MCF-7 cells were loaded into the cell chip and cultured for 24 h before treatment. Doxorubicin (TargetMol, Boston, MA, USA) solutions with a concentration gradient ranging from 0, 0.462, 4.62, 11.55, to 20.79 μM were dispensed into the cell chip using the NanoCLD, resulting in final concentrations of 0, 0.2, 2, 5, and 9 μM, respectively. After 24 h of incubation, the cell chip was gently washed three times with phosphate-buffered saline (PBS) and stained with calcein AM (green) and PI (red) for 20 min. Finally, the cell chip was submerged in a fresh culture medium within a Petri dish and was photographed by the IX83 microscope. The cell viability was calculated by manually counting the numbers of live cells (green) to the total cells (green and red) and plotted with the doxorubicin concentrations.

## 3. Results and Discussion

### 3.1. Nanoliter-Scale Liquid Handling

**Figure 4:**
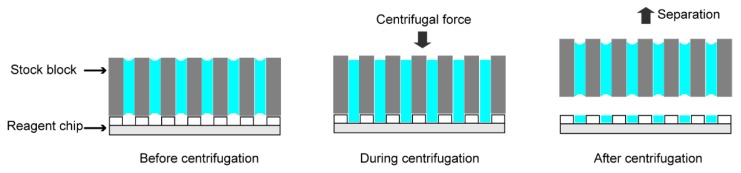
Liquid dispensing by centrifugation. Before centrifugation, the reagents are retained within the reagent holes of the stock block due to the capillary action. During centrifugation, the reagents are spun down into the microwells due to the centrifugal force and the excess air in the microwells is expelled out. After centrifugation, the stock block is taken out from the fixture and nanoliter-volume reagents are dispensed into the microwells.

The nanoliter centrifugal liquid dispenser utilizes the centrifugal force to load reagents into the microwells of the reagent chip. As illustrated in Figure 4, before centrifugation, the reagents are retained within the reagent holes of the stock block due to the capillary action and the air is in the microwells of the reagent chip. During centrifugation, the reagents are spun down into the microwells due to the centrifugal force and the excess air in the microwells is expelled through either the nanoporous superhydrophobic polymers or the reagent holes. Although the stock block and the reagent chip are just pressed together by the fixture, there is no leakage between these two parts mainly due to the superhydrophobic coating on both of the contact surfaces. After centrifugation, the stock block is firstly lifted out from the fixture. Due to restriction from the fixture, the separation of the stock block from the chip is achieved vertically, ensuring all the reagents to break apart almost simultaneously. As shown in Figure S2, while the red dye plugs were in different vertical positions in the reagent holes before dispensing, these plugs were all moved to the ends of the reagent holes after centrifugation, illustrating the effectiveness of this dispensing process.

**Figure 5:**
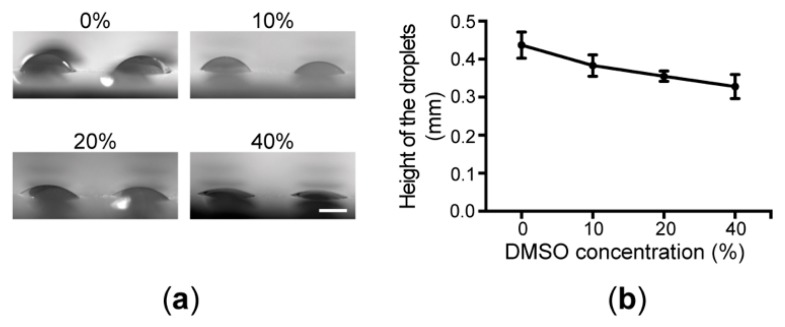
Nanoliter-volume liquid dispensing using the NanoCLD. (**a**) Side views of the dispensed droplets in microwells with different DMSO concentrations. (**b**) The correlation between the heights of the droplets and the DMSO concentrations (n = 20, scale bar: 400 μm).

The volume of dispensed liquid into the microwells is determined by four factors: (1) size of microwells of the reagent chip; (2) hydrophilic properties of the glass bottom of the reagent chip and the inner surfaces of the reagent holes; (3) surface tension of dispensed liquid; and (4) separation speed of the stock block from the reagent chip after centrifugation. In the current study, we tried to simplify the system to improve the reproducibility of liquid dispensing. Therefore, the sizes of the microwells were unchanged throughout the study so that the variation introduced by the chip fabrication was minimized. The calculated volumes of the microwells in the reagent and the cell chips were ~160 and 220 nL, respectively. The glass bottoms of the microwells and the inner surfaces of the reagent holes were left untouched to prevent the variations from any surface modification processes. The surface tension of the dispensed liquid determines how easy it is to break the liquid when the fixture is dissembled. As dimethyl sulfoxide (DMSO) is a common solvent for drug discovery applications, we employed DMSO solutions to evaluate the relationship between the surface tension and volumes of dispensed reagents. We discovered that when the DMSO concentration was over 50%, the superhydrophobic layer was destroyed, leading to cross-contamination. Therefore, we prepared a serial of binary mixtures of DMSO and water ranging from 0%, 10%, 20%, to 40% (DMSO in water, volume ratio). The surface tensions of these mixtures can be found as 71.6, 61.0, 56.0, and 52.5 mN/m at 25 °C, respectively, in the reference [30]. As shown in Figure 5, the dispensed volumes measured as the heights of the droplets (the measured height of the convex meniscuses plus 200 μm of the microwell height) did show a correlation with the surface tensions of the dispended mixtures, suggesting that we need to use a single solvent recipe for the entire analysis in future studies. Finally, the separation speed of the stock block from the reagent chip may also play a role in determining the size of dispensed liquid. Fortunately, due to the requirement of the alignment, the stock block was designed to be sliding-fitted into the fixture with a minimal clearance. As a result, the block can only be lifted out slowly by hand and no variation caused by this manual operation was observed (data not shown).

### 3.2. Characterization of the Liquid Dispensing

**Figure 6:**
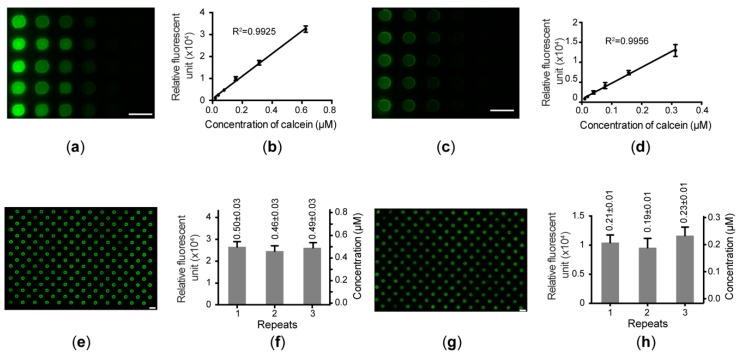
Quantitative characterization of nanoliter-volume liquid dispensing using the NanoCLD. (**a**) Fluorescent image of the microwells with a gradient of calcein dispensed into the reagent chip using the NanoCLD. (**b**) Linear relationship between the calcein concentrations and the fluorescence intensities of the microwells (n = 5). (**c**) Fluorescent image of the microwells with calcein delivered into the cell chip. (**d**) Linear calibration between the calcein concentrations and the fluorescence intensities (n = 5). (**e**) Chessboard pattern produced in the reagent chip using the NanoCLD. (**f**) Relative fluorescent units and corresponding calcein concentrations of microwells across the entire reagent chip in three independent repeats (n = 192 in each run). (**g**) Fluorescent image of the cell chip sandwiched with the reagent chip with the chessboard pattern. (**h**) Relative fluorescent units and converted calcein concentrations of microwells in the cell chip in three independent runs (n = 192 in each run). (scale bar: 1.5 mm).

The quantitative delivery of reagents into individual microwells across the entire chip without any cross-contamination is the prerequisite to utilizing this NanoCLD for high-throughput cell analysis. To quantitate the reagents being transferred in the dispensing process, we prepared serially diluted calcein solutions with concentrations of 0.02, 0.04, 0.08, 0.16, 0.3, and 0.6 μM. As shown in Figure 6a,b, calcein solutions were first loaded into the reagent chip with the NanoCLD, and the images of the microwells were taken under a microscope. The fluorescence intensities of the microwells showed a linear correlation with the calcein concentrations with a R^2^ of 0.99. Next, the reagent chip with the dispensed calcein was sandwiched to the cell chip, which was preloaded with DI water. The resulting calcein gradient in the chip assembly demonstrated a linear relationship with a R^2^ of 0.99 as well (Figure 6c,d). Both the linear results proved that the quantitative dispensing of reagents can be obtained with the NanoCLD.

To verify the reproducibility of the liquid dispensing by the NanoCLD, we loaded 0.5-μM calcein into the stock block in a chessboard pattern, followed by the centrifugal loading of calcein into a reagent chip and the transfer to a cell chip. The entire reagent chip and the chip assembly were scanned under the microscope to evaluate the uniformity of the loading using the measured fluorescence intensities. This operation was repeated three times independently to prove the run-to-run reproducibility. As shown in Figure 6e and 6f, the first dispensing to the reagent chip with the NanoCLD can produce the same chessboard pattern on the reagent chip without any cross contamination. The fluorescence intensities of the microwells with calcein in three repeats were measured to be 26513 ± 2356, 24589 ± 2389, and 26111 ± 2291, which correspond to 0.50 ± 0.03, 0.46 ± 0.03, and 0.49 ± 0.03 μM, respectively, based on the calibration curve in Figure 6b. The coefficients of variations (CVs) were all less than 7% in three runs. The run-to-run average concentration was 0.48 ± 0.02 μM with a CV of 4.3%. Next, the calcein droplets in the reagent chips were transferred to the cell chips, producing the same chessboard pattern shown in Figure 6g. The fluorescence intensities of the microwells were measured to be 10440 ± 1307, 9570 ± 1596, and 11642 ± 1464, which correspond to 0.21 ± 0.01, 0.19 ± 0.01, and 0.23 ± 0.01 μM, respectively (Figure 6h). These concentrations were calculated using the calibration curve shown in Figure S3. The quantitative analysis proved that the run-to-run CV was less than 10% (0.21 ± 0.02 μM). During the second transfer, calcein was diluted by water preloaded in the cell chips. The average dilution factor was calculated to be 2.31, which is roughly equal to the volumes ratio of 2.38 calculated based on the design (160 and 220 nL in the reagent and the cell chips, respectively). Therefore, the dilution factor of 2.31 was used in the rest of study to determine the concentration in the stock block in order to obtain a certain concentration in the final reaction.

### 3.3. Cell Culture on the Chip

**Figure 7:**
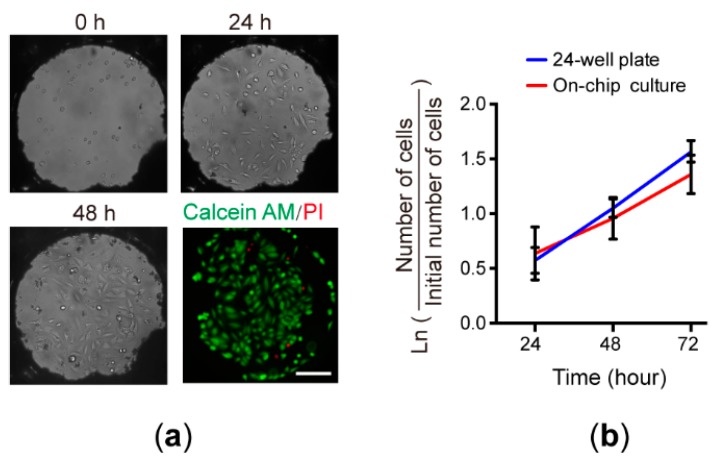
Cell culture on the chip. (**a**) Bright-field and fluorescent micrographs of MCF-7 cells cultured in individual microwells for three days (scale bar: 200 μm). After 72 h, cells were stained with calcein AM and propidium iodide, showing more than 93% of the cells were alive. (**b**) Comparison of the growth rates of MCF-7 cells cultured in the microwells (n = 20) and in the 24-well plate (n = 4).

In our previous study, we had successfully demonstrated the long-term culture of various cells—including BHK-21, passage 4 human umbilical vein endothelial cells (HUVECs), K562, and Oct4-EGFP mouse induced pluripotent stem cells (iPSCs)—on the superhydrophobic microwell array chip [22]. The average population doubling time and the viability of these cells in the microwells were similar to those in 24-well macroscale cultures. In the current study, during the drug treatment process, cells were cultured within the microwells covered with the reagent chip. This enclosed culture condition is different from that presented previously [22]. Therefore, here we reexamined whether cells could be cultured on the cell chip without sacrificing cell viability when the reagent chip was put in place. We first seeded MCF-7 cells into the microwells at a density of 50 ± 10 cells per wells. The assembled chip was placed in a covered Petri dish with excess sterile water surrounding the chip. The culture medium was changed every 12 h and the images of the microwells were taken under the microscope after each medium exchange. As shown in Figure 7a, all the cells in the microwells exhibited normal morphology throughout the three-day culture. At the end of the culture, cells were stained with calcein AM and propidium iodide, showing that more than 93% of the cells were alive. In addition, Figure 7b illustrates that the growth rate of the MCF-7 cells in the microwells was close to that in the conventional 24-well culture. These results prove that our new culture system can be used for cell assays without the negative influence of the on-chip culture condition.

### 3.4. Doxorubicin-Induced Cell Apoptosis

**Figure 8:**
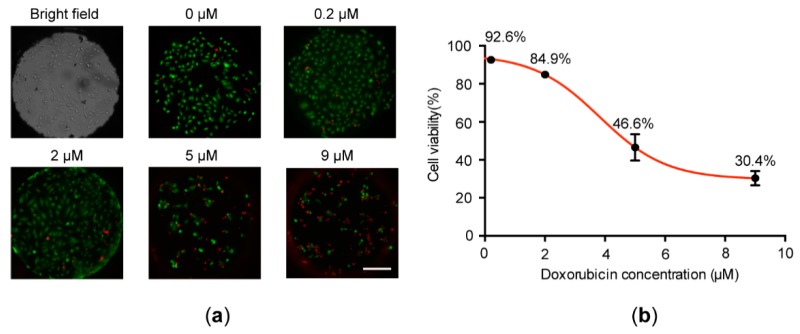
Doxorubicin-induced cell apoptosis on the chip. (**a**) Fluorescent micrographs of MCF-7 cells treated with different concentrations of doxorubicin and stained with calcein AM and PI (scale bar: 200 μm). (**b**) Calibration curve of cell viability vs. doxorubicin concentrations (n = 7).

After the verification of cell culture on the chip, we next employed doxorubicin—an antibiotic agent that inhibits DNA topoisomerase II and induces DNA damage and apoptosis [31]—to test the feasibility of dispensing reagents with the NanoCLD for high-throughput cell analysis. First, about 100 MCF-7 cells were seeded into the cell chip. After cells were attached to the bottom of the microwells in a 24 h incubation, a gradient of doxorubicin diluted in culture medium together with a pure medium as a blank control was dispensed to cells to induce cell apoptosis. During the 24 h drug incubation, we did not perform the medium exchange to prevent dead cells from being washed away. As the cell confluency was less than 70%, we did not observe cell overlap occurred in the microwells. As shown in Figure 8a, when no doxorubicin was introduced, the cell viability was maintained at a level of 93%. With the increase of the concentration from 0.2, 2, 5, to 9 μM, the corresponding cell viabilities were decreased from 92.6%, 84.9%, 46.6%, and 30.4%. The fitted curve shown in Figure 8b perfectly reproduced the similar S curve reported in the literature. Although the current experiment is just a proof of concept in a small scale, it validates the feasibility of performing high-throughput cell-based assays on our platform in the future.

## 4. Conclusions

We have developed a novel nanoliter centrifugal liquid dispenser for conveniently introducing nanoliter reagents into microwell arrays in a high-throughput manner. With a simple centrifugation, 384 droplets can be delivered into microwells simultaneously. This quantitative, uniform, and reproducible delivery of reagents has been thoroughly characterized to prove the feasibility of performing cell-based assays on our system. Previously, we had demonstrated a cell chip with over 1000 microwells arranged in an area of a glass slide [22]. By covering multiple reagent chips onto this cell chip, a higher throughput can be easily achieved without any changes to the NanoCLD system. In the current study, the reagents were still manually loaded into the stock block from 96-well plates. However, a stock block with 384 reagent holes can easily be loaded by pipetting 48 times using an 8-channel adjustable tip spacing pipette. As the stock block can be used as a container for reagent storage, the reagents in the stock block can be saved up to several months and dispensed out whenever needed for over twenty times. Therefore, the NanoCLD is more useful for repeatedly dispensing reagents in common laboratories that do not have access to high-end robotic workstations, and a lot of pipetting labors can be saved by our system. In future, the dispensing accuracy can be further improved by standardizing the sizes of the array chips and by optimizing the structure and the operation of the fixture. In addition, more types of targets, such as cells or beads, can be dispensed into microwells using the NanoCLD for broader applications.

## Supplementary Materials

The following are available online at http://www.mdpi.com/2072-666X/9/6/286/s1, Figure S1: Photo of stock blocks wrapped with Parafilm M for storage and without film; Figure S2: Plugs of Rhodamine B dye loaded in reagent holes of the stock block before and after centrifugation; and Figure S3: Calibration curve between the calcein concentrations and fluorescence intensities in the reagent and cell chip assembly; Video S1: The operation of NanoCLD.
