# Supplementary material for: Nanoliter Centrifugal Liquid Dispenser Coupled with Superhydrophobic Microwell Array Chips for High-Throughput Cell Assays
Source: Micromachines (Basel). 2018 Jun 6;9(6):286. doi: 10.3390/mi9060286 (PMC6187582; doi:10.3390/mi9060286)
Supplement: Supplementary file 1 [file micromachines-09-00286-s001.zip › Supplementary/YWang_supplementary_Micromachines.pdf]

Supplementary Information

Nanoliter centrifugal liquid dispenser coupled with superhydrophobic microwell array chips for high-throughput cell assays

Yuyi Wang[1, #], Yushuai Wu[1, #], Yue Chen[1], Jianxiong Zhang[1], Xiaofang Chen[2] and Peng Liu[1,*]

[1] Department of Biomedical Engineering, School of Medicine, Collaborative Innovation Center for Diagnosis and Treatment of Infectious Diseases, Tsinghua University, Beijing, 100084, China
[2] School of Biological Science and Medical Engineering, Beihang University, Beijing, 100191, China

# Joint first authors with equal contributions
* Correspondence: pliu@tsinghua.edu.cn; Tel.: +86-10-62798732

Supplementary Figures

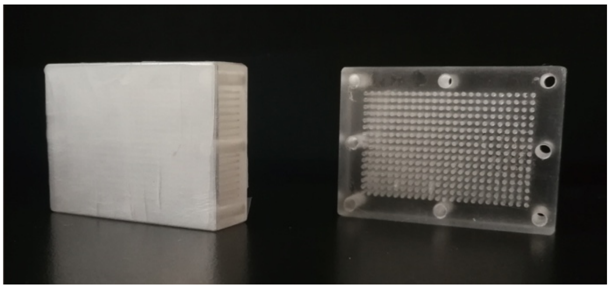

Figure S1. Photo of stock blocks wrapped with Parafilm M for storage (left) and without film (right).

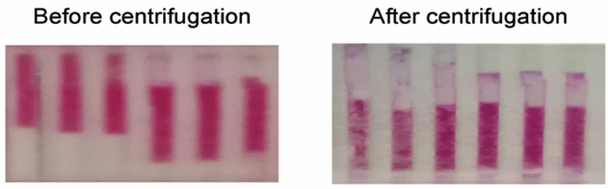

Figure S2. Plugs of Rhodamine B dye loaded in reagent holes of the stock block before and after centrifugation.

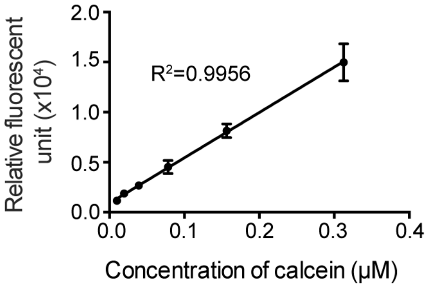

Figure S3. Calibration curve between the calcein concentrations and fluorescence intensities in the reagent and cell chip assembly (n=5). For each calcein concentration, the calcein solution was loaded into the reagent chip by NanoCLD, and the cell chips submerging the entire chips into the same concentration of calcein solution followed by aspirating away the excess. After that, the reagent and the cell chips were sandwiched together face-to-face for imaging.
