# Supplementary figures and images for: Nanoliter Centrifugal Liquid Dispenser Coupled with Superhydrophobic Microwell Array Chips for High-Throughput Cell Assays
Source: Micromachines (Basel). 2018 Jun 6;9(6):286. doi: 10.3390/mi9060286 (PMC6187582; doi:10.3390/mi9060286)

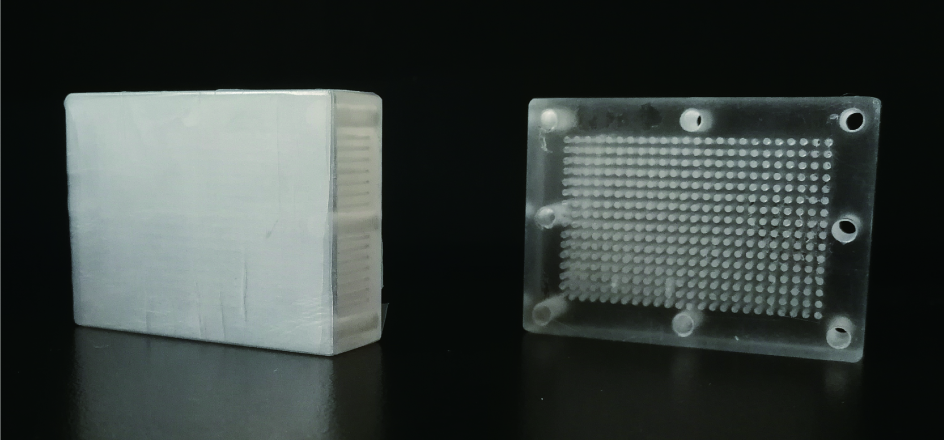

Supplement: Supplementary file 1 [file micromachines-09-00286-s001.zip › Supplementary/Figure S1.tif]

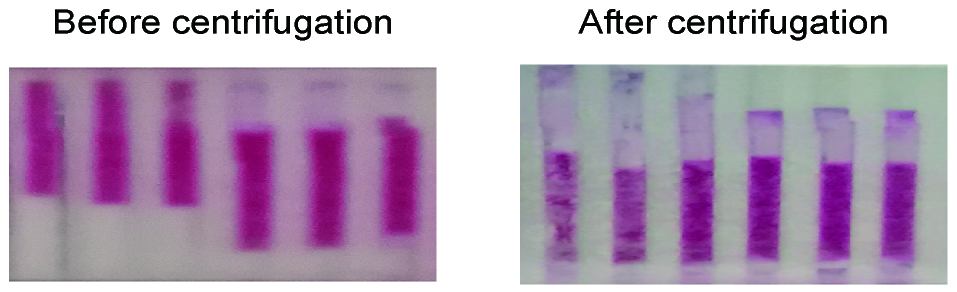

Supplement: Supplementary file 1 [file micromachines-09-00286-s001.zip › Supplementary/Figure S2.tif]

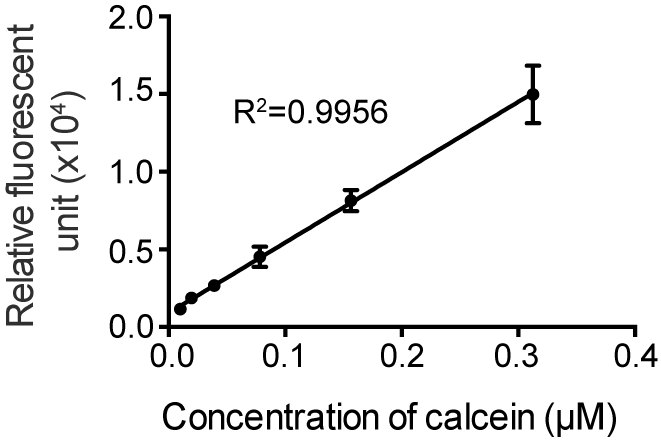

Supplement: Supplementary file 1 [file micromachines-09-00286-s001.zip › Supplementary/Figure S3.tif]
